# Engineering of 3-ketosteroid-∆^1^-dehydrogenase based site-directed saturation mutagenesis for efficient biotransformation of steroidal substrates

**DOI:** 10.1186/s12934-018-0981-0

**Published:** 2018-09-10

**Authors:** Shuhong Mao, Jian-Wen Wang, Fufeng Liu, Zhangliang Zhu, Dengke Gao, Qianqian Guo, Panpan Xu, Zheng Ma, Yali Hou, Xiaotao Cheng, Dengyue Sun, Fuping Lu, Hui-Min Qin

**Affiliations:** 1 State Key Laboratory of Food Nutrition and Safety, Tianjin, People’s Republic of China; 2 Key Laboratory of Industrial Fermentation Microbiology, Ministry of Education, Tianjin, People’s Republic of China; 3 Tianjin Key Laboratory of Industrial Microbiology, Tianjin, People’s Republic of China; 4 College of Biotechnology, Tianjin University of Science and Technology, Tianjin, People’s Republic of China; 5 National Engineering Laboratory for Industrial Enzymes, Tianjin, 300457 People’s Republic of China

**Keywords:** Dehydrogenase, Saturation mutagenesis, Rational design, Steered molecular dynamics, 3-ketosteroid

## Abstract

**Background:**

Biosynthesis of steroidal drugs is of great benefit in pharmaceutical manufacturing as the process involves efficient enzymatic catalysis at ambient temperature and atmospheric pressure compared to chemical synthesis. 3-ketosteroid-∆^1^-dehydrogenase from *Arthrobacter simplex* (KsdD3) catalyzes 1,2-desaturation of steroidal substrates with FAD as a cofactor.

**Results:**

Recombinant KsdD3 exhibited organic solvent tolerance. W117, F296, W299, et al., which were located in substrate-binding cavity, were predicted to form hydrophobic interaction with the substrate. Structure-based site-directed saturation mutagenesis of KsdD3 was performed with W299 mutants, which resulted in improved catalytic activities toward various steroidal substrates. W299A showed the highest increase in catalytic efficiency (*k*_*cat*_/*K*_m_) compared with the wild-type enzyme. Homology modelling revealed that the mutants enlarged the active site cavity and relieved the steric interference facilitating recognition of C17 hydroxyl/carbonyl steroidal substrates. Steered molecular dynamics simulations revealed that W299A/G decreased the potential energy barrier of association of substrates and dissociation of the corresponding products. The biotransformation of AD with enzymatic catalysis and resting cells harbouring KsdD3 WT/mutants revealed that W299A catalyzed the maximum ADD yields of 71 and 95% by enzymatic catalysis and resting cell conversion respectively, compared with the wild type (38 and 75%, respectively).

**Conclusions:**

The successful rational design of functional KsdD3 greatly advanced our understanding of KsdD family enzymes. Structure-based site-directed saturation mutagenesis and biochemical data were used to design KsdD3 mutants with a higher catalytic activity and broader selectivity. 
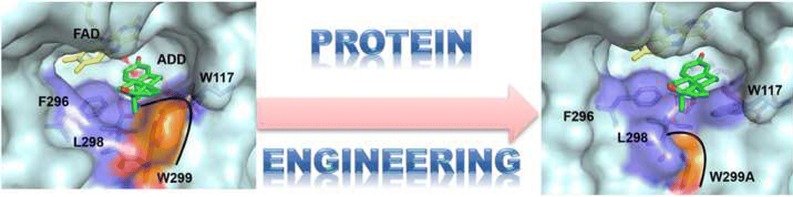

**Electronic supplementary material:**

The online version of this article (10.1186/s12934-018-0981-0) contains supplementary material, which is available to authorized users.

## Background

Biotransformation provides an alternative method for the production of steroidal medicine intermediates by employing enzymatic processes that are “green” and efficient compared with chemical synthesis [1, 2]. Various microorganisms can be exploited for the synthesis of desirable steroids, and the large diversity of metabolic reactions available represents a rich repository of chemical modification [3–8]. Compared to chemical synthesis, biotransformation of steroidal drugs requires no multi-step protection/deprotection, high-pressure, the use and removal of precious and toxic transition metals, or some reagents, i.e. pyridine, sulfur trioxide or selenium dioxide, which increase environmental burden [9–13]. For instance, *Arthrobacter simplex* has been used for steroid hydroxylation, dehydrogenation and sterol side-chain cleavage because of its high tolerance to organic solvents and remarkable bioconversion [14–16], involving the ∆^1^-dehydrogenation of steroids. This dehydrogenation is a crucial modification in steroid synthesis because it increases the biological activity and economical value of the original steroidal substrate [17, 18]. Both cortisone acetate and prednisone acetate are used as anti-inflammatory and anti-allergy drugs clinically [14, 19, 20]. The anti-inflammatory activity of prednisolone acetate increased three- to fourfold when introducing a C1–C2 double bond into ring A of hydrocortisone acetate by ∆^1^-dehydrogenation, catalyzed by 3-ketosteroid-∆^1^-dehydrogenase [14, 21, 22].

**Fig. 1:**
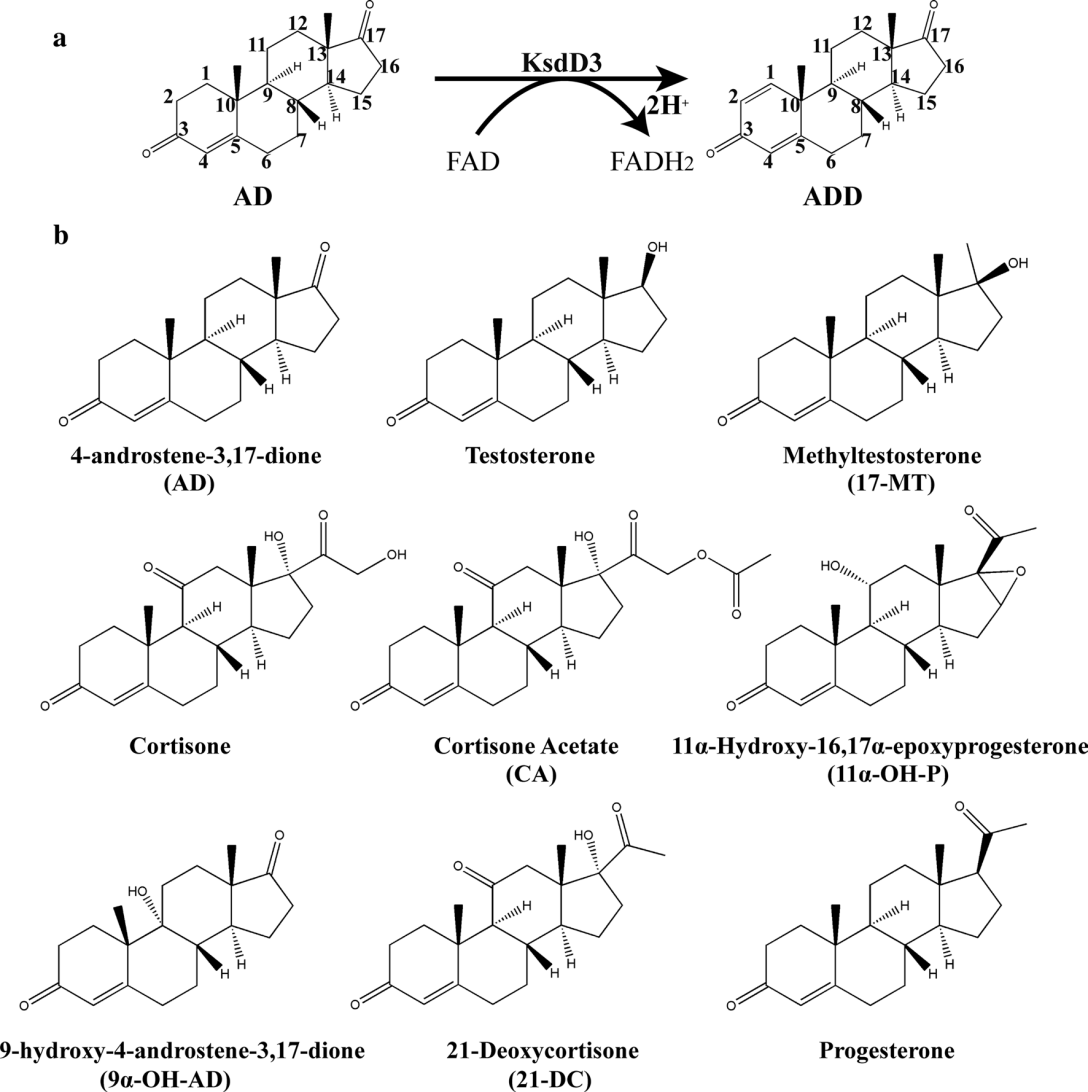
**a** KsdD3 catalyzes the dehydrogenation at C1,2-position of steroidal substrates. **b** Nine substrate structures of steroids are used to determine the substrate specificity of KsdD3

3-Ketosteroid-∆^1^-dehydrogenase (KsdD, EC 1.3.99.4), which catalyzes the insertion of a double bond between the C1 and C2 atoms of the 3-ketosteroid A-ring (Fig. 1a), has been found in several steroid-degrading bacteria, including *A. simplex* [22], *Rhodococcus rhodochrous* [23], *Pseudomonas testosteroni* [24], *Nocardia coralline* [25], and *Mycobacterium* sp. [26]. The enzyme KsdD is a flavoprotein dehydrogenating a wide variety of 3-ketosteroids. It is a key enzyme in microbial steroid catabolism needed for opening of the steroid B-ring. The catalytic mechanism of dehydrogenation of the C1–C2 bound of 3-ketosteroids has been elucidated: the two hydrogen atoms on the respective C1 and C2 atoms of the substrate undergo direct elimination without the formation of an hydroxyl intermediate or H_2_O [27–29]. A two-step mechanism has been proposed that indicates that the catalytic process proceeds via trans-diaxial elimination of the 1α,2β hydrogen atoms from a 3-ketosteroid substrate [30]. Interaction of the C3 carbonyl group of a steroidal substrate with an electrophile promotes labilization of the hydrogen atoms at the C2 position. A general base has been proposed to abstract a proton from C2 atom resulting in an enolate or a carbanionic intermediate. After hydride ion transfer from the C1 atom to flavin adenine dinucleotide (FAD) [31, 32], a double bond is then formed between the C1–C2 atoms. The crystal Structure of KstD1 from *Rhodococcus erythropolis* revealed that Tyr487 and Gly491 promoted tautomerization, and Tyr318/Tyr119 and FAD abstracted a proton and a hydride ion, respectively [33, 34]. These researches provided a fundamental understanding of KstD family enzyme, and the theoretical support for rational design of *A. simplex* KsdD3.

Recently, a novel putative 3-ketosteroid-∆^1^-dehydrogenase gene from *A. simplex* (KsdD3, also called *Pimelobacter simplex*) has been identified. KsdD3 contained 1548 bp of a complete open reading frame encoding a protein (516 amino acids, 54.3 kDa) without signal peptide. Efforts have been made on *A. simplex* 156 to improve the ∆^1^-dehydrogenation efficiency of this dehydrogenase by genetic manipulation, in which the control of the cat promoter was transferred into the strain and integrated into the 16S rDNA sites [14]. In our preparing experiment, we found that KsdD3 was largely overexpressed in solubility, and it showed the high catalytic activity. However, the enzymatic characterization and catalytic activity of KsdD3 remain unclear, and the mechanism of substrate recognition and selectivity are poorly understood. This has hampered its further application as an industrial catalyst. Therefore, the rational design of functional KsdD3 based on structural modelling analysis would strengthen our understanding of KsdD family enzymes [35]. To engineer an enzyme with a higher catalytic activity and broader selectivity, structure-based site-directed saturation mutagenesis and biochemical assays were used to obtain KsdD3 mutants.

## Methods

### Materials

All chemicals and reagents used in this study were at least analytical grade or better. Phenazine methosulphate (PMS) and FAD were purchased from Solarbio (Beijing, China). 6-dichlorophenolindophenol (DCPIP), AD, ADD, testosterone, boldenone, methyltestosterone and metandienone were purchased from YuanYe (Shanghai, China). Cortisone, prednisone, cortisone acetate (CA), prednisone acetate (PA), 11α-hydroxy-16,17α-epoxyprogesterone, 9α-OH-AD, 21-deoxycortisone, progesterone and 1-dehydroprogesterone were supplied by J&K Scientific Co., Ltd (Beijing, China). KOD-Plus-Mutagenesis Kit was purchased from Toyobo Life Science (Shanghai, China). PrimeSTAR MAX DNA polymerase, Restriction endonucleases, T4 DNA ligases was purchased from TaKaRa (Dalian, China). Synthetic oligonucleotides were purchased from GENEWIZ (Beijing, China). All standard reagents were purchased from Aldrich/Sigma if not stated otherwise.

### Cloning, expression and purification

The genomic DNA of *A. simplex* 156 was used as a template. The KsdD3 gene (GenBank accession No. AIY19527.1; Protein ID: WP_038682818.1) was amplified using PrimeSTAR MAX as DNA polymerase. The KsdD3 gene was subsequently inserted into pET28a(+) (Novagen, Madison, WI, USA) between the *Nde* I and *Eco*R I sites with a His_6_ tag (HHHHHH) at the N-terminus. *E. coli* BL21(DE3) harboring pET28a-KsdD3 was grown in Luria–Bertani (LB) medium at 37 °C and 160 rpm. When the OD_600_ reached 0.6–0.8, 0.5 mM isopropyl β-d-1-thiogalactopyranoside (IPTG) was added into the cultures, and the medium was further incubated at 16 °C for 20 h. The cells were harvested by centrifugation at 5000×*g* and 4 °C for 15 min, and were then resuspended in lysis buffer [20 mM Tris–HCl (pH 8.0), 20 mM imidazole, 0.5 M NaCl, and 1 mM dithiothreitol (DTT)]. The pellets were disrupted by sonication, and centrifuged at 40,000×*g* for 30 min at 4 °C for the removal of cell debris. KsdD3 proteins in the supernatant were trapped on Ni–NTA superflow resin (Qiagen, Hilden, Germany). After washing, KsdD3 was eluted with elution buffer [20 mM Tris–HCl (pH 8.0), 400 mM imidazole, 0.3 M NaCl, and 1 mM DTT]. The KsdD3 was identified with 98% purity on SDS-PAGE with Image Lab™ Software version 5.2.1 (Bio-Rad, Hercules, CA, USA) (Additional file 1: Fig. S1). The yellow-coloured KsdD3 was exchanged into buffer [20 mM Tris–HCl (pH 8.0), 0.2 M NaCl and 20 µM FAD] via ultrafiltration (10 kDa molecular weight cutoff), finally diluted with the same buffer to 5 mg L^−1^ for enzyme activity assay. Protein concentrations were determined using the BCA protein assay kit (Solarbio, Beijing), with bovine serum albumin as a standard [36, 37].

### Activity assay

The enzyme activities of KsdD3 and its mutants were determined spectrophotometrically at 30 °C using PMS and DCPIP as electron acceptors [34, 38]. The reaction mixture consists of 50 mM Tris–HCl, pH 7.5, 1.5 mM PMS, 40 μM DCPIP and 500 μM AD in methanol (2%) in 1 mL volume. The reaction was initiated by adding purified KsdD3 to a final concentration of 3.1 μM. The control was prepared by using the reaction system without the KsdD3 enzyme. In the dehydrogenation reaction, two protons were transferred from FADH_2_ to form PMSH_2_, and the reduction of DCPIP was performed by transferring 2H of PMSH_2_ to form DCPIPH_2_, which caused a decrease in the absorbance at 600 nm. The decreased absorbance at 600 nm (ε_600_ nm = 18.7 × 10^3^ cm^−1^ M^−1^) was monitored using a microplate reader [Infinite M200 Pro (Tecan Austria GmbH, Austria)] every 20 s for 3 min. One unit of activity was defined as the amount of enzyme giving a reduction of 1 µmol min^−1^ DCPIP. Specific activities are defined as nmol mg^−1^ min^−1^ (mU mg^−1^).

Nine substrates, such as 4-androstene-3,17-dione (AD), testosterone, methyltestosterone (17-MT), cortisone, cortisone acetate (CA), 11α-hydroxy-16,17α-epoxyprogesterone (11α-OH-P), 9α-hydroxy-4-androstene-3,17-dione (9α-OH-AD), 21-deoxycortisone (21-DC), and progesterone were used to determine the substrate selectivity of KsdD3 (Fig. 1b). The reaction systems are the same as used in the method described above. All experiments were repeated three times, and the data are shown as mean ± SD.

### Characterization of KsdD3

AD was used as the substrate for the characterization of KsdD3. The optimal pH for purified KsdD3 was determined at 30 °C using different reaction buffers, which contained 50 mM each of MES, PBS, HEPES, Tris–HCl and Gly–NaOH in the pH rang of 5.5–9.5. KsdD3 activity at 30 °C, Tris–HCl buffer pH 7.5 was defined as 100%.

The effect of pH on the stability of KsdD3 was measured by determination of the residual activity in the standard assay, after buffer exchange of KsdD3 and pre-incubation for 2 h at 4 °C. Buffer exchange was performed by ultrafiltration (Millipore, Merck, German) at 4 °C and 3000×*g.* The maximal KsdD3 activity at 30 °C, Tris–HCl buffer pH 8.0 was defined as 100%.

To determine the optimal temperature, the activity assay was performed at various temperatures from 25 to 55 °C in 50 mM Tris–HCl buffer (pH 7.5). KsdD3 activity at 30 °C, Tris–HCl buffer pH 7.5 was defined as 100%.

The thermostability was determined at 30 °C by measuring the residual activity, after the incubation of the enzyme in Tris–HCl buffer (50 mM, pH 8.0) at different temperatures (25–55 °C) for 30, 60 and 120 min, and then cooling the mixture on ice for 10 min. The maximal KsdD3 activity at 25 °C, Tris–HCl buffer pH 8.0 was defined as 100%.

The effect of the organic solvents (dissolving the same amount of AD) on KsdD3 activity was determined by adding different volumes (0.8–35%, v/v) of methanol into the reaction system. The maximal KsdD3 activity at 30 °C, Tris–HCl buffer pH 7.5 and 2% methanol was defined as 100%.

To measure the organic-solvent tolerance, KsdD3 was pre-incubated in various solvents (methanol, ethanol, isopropyl alcohol, DMSO, DMF, acetone and acetonitrile) at different concentrations (10–50%, v/v) for 2 h in Tris–HCl buffer (50 mM, pH 8.0) at 4 °C. Before determination, the mixture was cooled on ice for 10 min, and the activity was then measured under standard reaction conditions of 30 °C and Tris–HCl buffer pH 8.0. The maximal KsdD3 activity under standard reaction conditions was defined as 100%.

Kinetic studies of KsdD3 wild-type (WT) and mutants were performed in the same reaction system using seven different concentrations of steroidal substrates, i.e., CA and 11α-OH-P: 0–300 μM; 21-DC: 0–400 μM; others: 0–500 μM. The fitting curves of KsdD3 WT and mutants toward seven steroidal substrates were plotted, and the kinetic parameters, *K*_m_ and *k*_cat_ were calculated using the Michaelis–Menten equation with GraphPad Prism 7.0 (GraphPad software, La Jolla, CA) by employing nonlinear regression.

$$ {\text{V}} = \frac{{{\text{V}}_{{\text {max} }}  \cdot [{\text{S}}]}}{{K_{\text{m}} + [{\text{S}}]}}\quad k_{\text{cat}} = \frac{{{\text{V}}_{{\max }} }}{C} $$

where V is the substrate consumption rate in µmol s^−1^ L^−1^; *V*_max_ is the maximum substrate consumption rate; [S] is the substrate concentration in µM; C is the concentration of KsdD3 in the reaction mixture; *K*_m_ is the Michaelis–Menten constant in µM, and is equal to the concentration of the substrate when the reaction rate is half of the maximum velocity; *k*_cat_ represents the turnover number in s^−1^, and *k*_cat_/*K*_m_ is also a constant on behalf of the catalytic efficiency of KsdD3 WT and mutants.

### Product analysis of steroidal substrates using HPLC, GC–MS and NMR

The reaction system contains 10 mg of KsdD3, Tris–HCl buffer (50 mM, pH 8.0), 2 mM DTT, 500 µM steroids in DMSO (2%), and 10 µM FAD. Reactions were incubated at 30 °C overnight and then terminated by boiling water at 100 °C for 5 min. Denatured protein was removed by centrifugation at 12,000 rpm for 10 min. The reaction products were extracted with 0.5 mL of ethyl acetate, twice. After drying under a stream of nitrogen gas, the steroidal products were resuspended in 20 μL methanol for HPLC and GC–MS analysis, or dissolved in deuterated chloroform/DMSO for NMR analysis.

The products of 1,4-androstadiene-3,17-dione, boldenone, methandienone, prednisone, prednisone acetate and 1-dehydroprogesterone were analyzed by GC–MS (VARIAN 4000 GC/MS) using a HP-5MS column (30 m × 0.25 mm × 0.25 mm, Agilent Technologies) in electron ionization (70 eV) mode. The temperature programming for GC column is the same as published before, and helium was used as the carrier gas at a flow rate of 1 ml min^−1^ [37]. The retention times and mass spectra of all peaks obtained were compared with those of standards available in the NIST library (nistmasspeclibrary.com).

The products of 16α,17α-epoxypregna-11α-hydroxy-1,4-diene-3,20-dione, 3-hydroxy-9,10-secoandrosta-1,3,5(10)-triene-9,17-dione and 17α-hydroxypregna-1,4-diene-3,11,20-trione were confirmed using NMR at 25 °C in deuterated chloroform or DMSO. The ^1^H and ^13^C NMR spectra were recorded on a Bruker AV-500 spectrometer at working frequencies 400 and 100 MHz, respectively. Chemical shifts (∆) are expressed in ppm values and coupling constants (*J*) in Hz. All spectra were processed using Bruker’s Topspin software using standard parameters.

### Site-directed mutagenesis

Site-directed mutagenesis of KsdD3 was performed by reverse PCR with pET28a-KsdD3 as a template using the KOD-Plus-Mutagenesis kit (Toyobo, Japan) [39]. The primers used for mutagenesis are summarized in Additional file 1: Table S1. PCR was conducted using temperature settings of 94 °C for 2 min followed by 8 cycles of 98 °C for 10 s, 68 °C for 8 min. The template plasmid was digested using *Dpn* I after PCR, and product was further cyclized by T4 polynucleotide kinase and ligation high (a DNA ligase). All KsdD3 mutants were expressed and purified using the same procedures as for the WT enzyme.

### Structure modeling of KsdD3

The three-dimensional (3D) model of KsdD3 was generated using Modeller 9.9.2 [40]. The crystal structure of 3-ketosteroid-∆^1^-dehydrogenase from *R. erythropolis* SQ1 (PDB ID: 4C3×, 2.0 Å, 46% sequence identity with KsdD3) was chosen as the template [34]. Homology modelling was performed by the automodel command. Thereafter, each model was optimized by the variable target function method with conjugate gradients. Simulated annealing MD was then used to refine the structure [41]. The best model was chosen based on the values of the Modeller objective function and the DOPE assessment scores.

### Steered molecular dynamics simulations and PMF calculations

Steered molecular dynamics (SMD) simulations apply an external force to the substrates’ center of mass that is pulled out from the KsdD3 active site along a predefined direction. The substrate-protein complexes were used in Gromacs 5.1.2 software using the Gromos 96 53A6 force field. The GROMOS96 53a6 force field parameters of the substrate were obtained from the Automated Topology Builder and Repository 2.0 webserver (https://atb.uq.edu.au/). The energy minimizations of the simulation systems including water molecules with and without substrate AD were optimized (Additional file 1: Table S2), and the potential energies of the two systems are shown in Table 2. The constant-velocity SMD simulations were performed in the present simulations. The pulling velocity was set to 0.01 Å ps^−1^. A spring constant of 1000 kcal mol^−1^ Å^−2^ was applied to the substrate’s centre of mass. Based on the above SMD trajectories, snapshots were taken to generate the starting configurations for the umbrella sampling windows. An asymmetric distribution of sampling windows was used to calculate the potential of mean force profiles of the WT and mutants over the distance along the substrate channel.

### Bioconversion of steroids with purified KsdD3 enzyme and resting cells of *E. coli*

Enzymatic conversion of steroids was carried out in a 20 mL reaction mixture consisting of 50 mM Tris–HCl (pH 8.0), 25 µg purified KsdD3, 25 µM FAD, 1 g L^−1^ substrates (dissolved in 3% v/v DMSO) and 0.1 mM PMS at 30 °C. Recombinant *E. coli* BL21(DE3) harboring KsdD3 were cultivated as described above. Cells were harvested and washed with 50 mM Tris–HCl (pH 8.0). The biotransformation was performed in 10-mL reaction volume (30 mg of cell wet weight mL^−1^) containing 50 mM Tris–HCl (pH 8.0), 0.5 mM PMS and 5 g L^−1^ substrates (dissolved in 5% v/v DMSO). 1.0 mL samples of resting cells were acquired at intervals. Cells were disrupted by sonication, and the products were extracted twice with ethyl acetate. After drying under a stream of nitrogen gas, the samples were resuspended in 1.0 mL methanol for HPLC analysis. All reactions with selected steroids were performed in triplicate with three independent measurements, and controls were prepared with *E. coli* BL21(DE3) not harboring KsdD3. The theoretical conversion of WT was defined as 100% for calculating the relative activity of the mutants.

HPLC (Agilent Technologies, Waldbronn, Germany) was used to separate the substrates and products using a Diamensil C18 column (5 μm, 4.6 × 250 mm) at 30 °C. The solvent systems and UV absorbance wavelengths used depended on the substrates: the mobile phase consisted of 70% methanol and 30% water (v/v) with a flow rate of 0.6 mL min^−1^ at 241 nm for the substrates AD, testosterone and 17-MT; 30% acetonitrile and 70% water (v/v) with a flow rate of 0.6 mL/min at 254 nm for cortisone [17]; 58% methanol and 42% water (v/v) with a flow rate of 1 mL min^−1^ at 240 nm for CA. The quantitative analysis of the target compounds was based on the standard curve (correlation coefficients were > 0.999). The yields of KsdD3 WT and mutants towards different steroids were calculated according to the equation:

$$ {\text{Yield }}\delta \, = \,\left( {{{{\text{C}}_{0} } \mathord{\left/ {\vphantom {{{\text{C}}_{0} } {{\text{C}}_{ 1} }}} \right. \kern-0pt} {{\text{C}}_{ 1} }}} \right)\, \times \, 100\% $$

where C_0_ and C_1_ are the concentration of the experimental and theoretical products, respectively.

## Results and discussion

### Enzyme characteristics of KsdD3

**Fig. 2:**
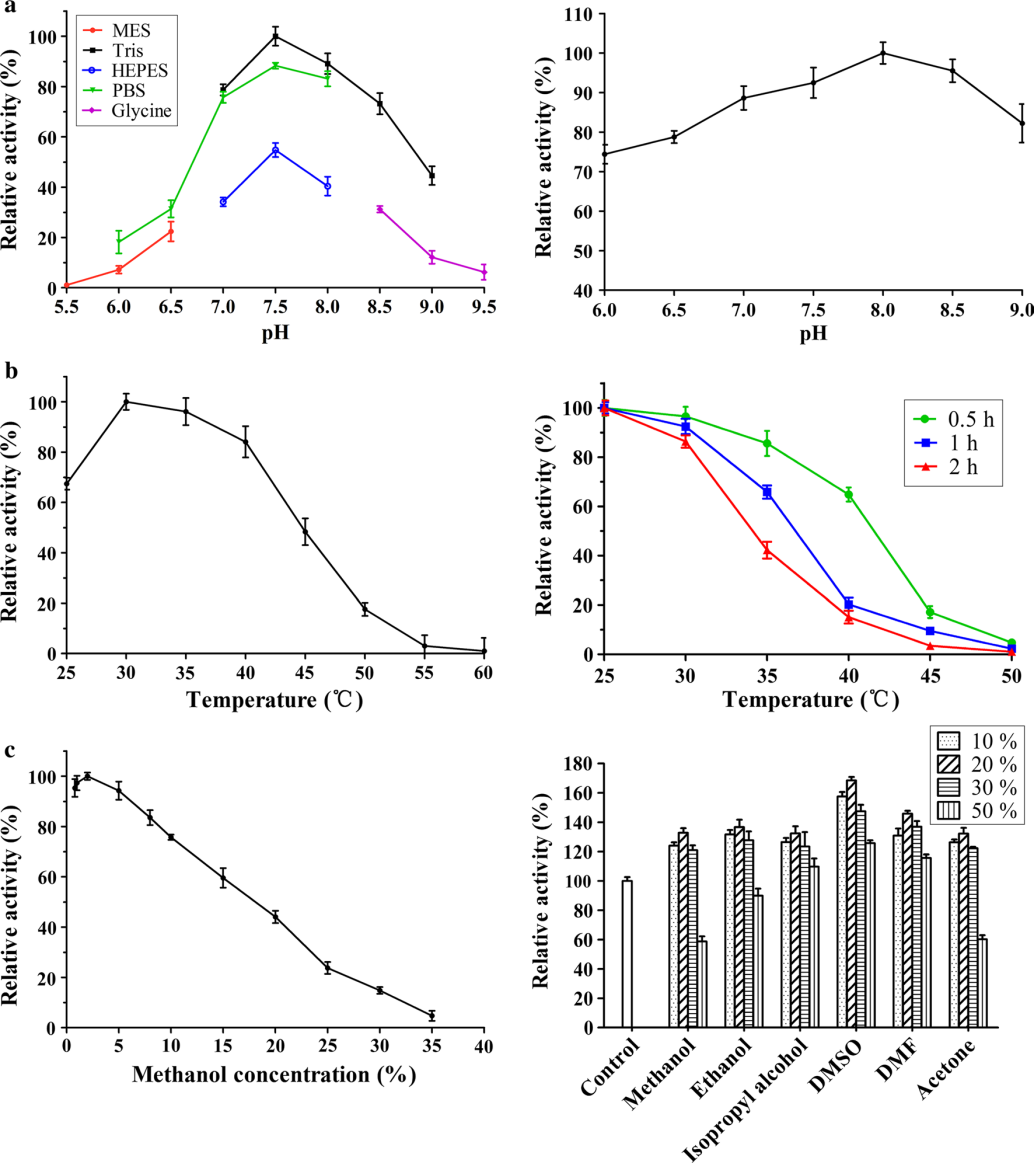
Effect of pH, temperature, organic solvents on activity of KsdD3. **a** pH dependence (Left) and pH stability (Right) of KsdD3. **b** Temperature dependence (Left) and thermostability analysis (Right) of KsdD3. **c** Effect of methanol concentration on reaction conditions was measured (Left). Organic solvents were added to the solution at 4 °C for 2 h (Right)

KsdD3 is a FAD-dependent enzyme that catalyzes the formation of a double bond at the C1–2 position of 3-ketosteroid substrates (Fig. 1a). The catalytic activity of KsdDs is highly dependent on the assay conditions [17, 27], and a comparison of buffer composition, pH, temperature and surfactant used to solubilize the substrate was investigated. The purified WT KsdD3 was active at pH 6.0–9.5 and the maximal activity was observed in Tris–HCl buffer pH 7.5. The KsdD3 exhibited good pH stability over the pH range 7.0–9.0 and more than 85% of the maximal activity was retained over this range (Fig. 2a). The optimal temperature for KsdD3 activity was 30 °C. The activity began to decrease dramatically when the temperature was over 40 °C. KsdD3 showed a better thermostability after incubating the enzyme in Tris–HCl buffer at different temperatures (25–55 °C) for 0.5 h, which retained over 65% residual activity until 40 °C. However, there was only 14% residual activity after pre-incubation at 40 °C for 1–2 h (Fig. 2b). KsdD3 lost almost all the activity at 45 °C. 3-ketosteroid-∆^1^-dehydrogenase catalyzes the reaction of substrates in organic solvents and detergents using hydrophobic interactions because of the negligible solubility of steroidal substrates in buffers. KsdD3 retained over 80% activity in reaction systems including up to 7.5% (v/v) methanol, but lost almost all catalytic ability in 35% methanol. The catalytic activity of KsdD3 in various micelles was determined to investigate organic solvent resistance. All micelle solutions improved enzyme activity when KsdD3 was treated with 10–30% solutions, indicating that KsdD3 from *A. simplex* exhibited a higher tolerance to other organic solvents than for methanol (Fig. 2c). This is consistent with the previous reports that *A. simplex* has been widely used in steroidal transformations for its high tolerance to organic solvents [42].

### Substrate selectivity

**Fig. 3:**
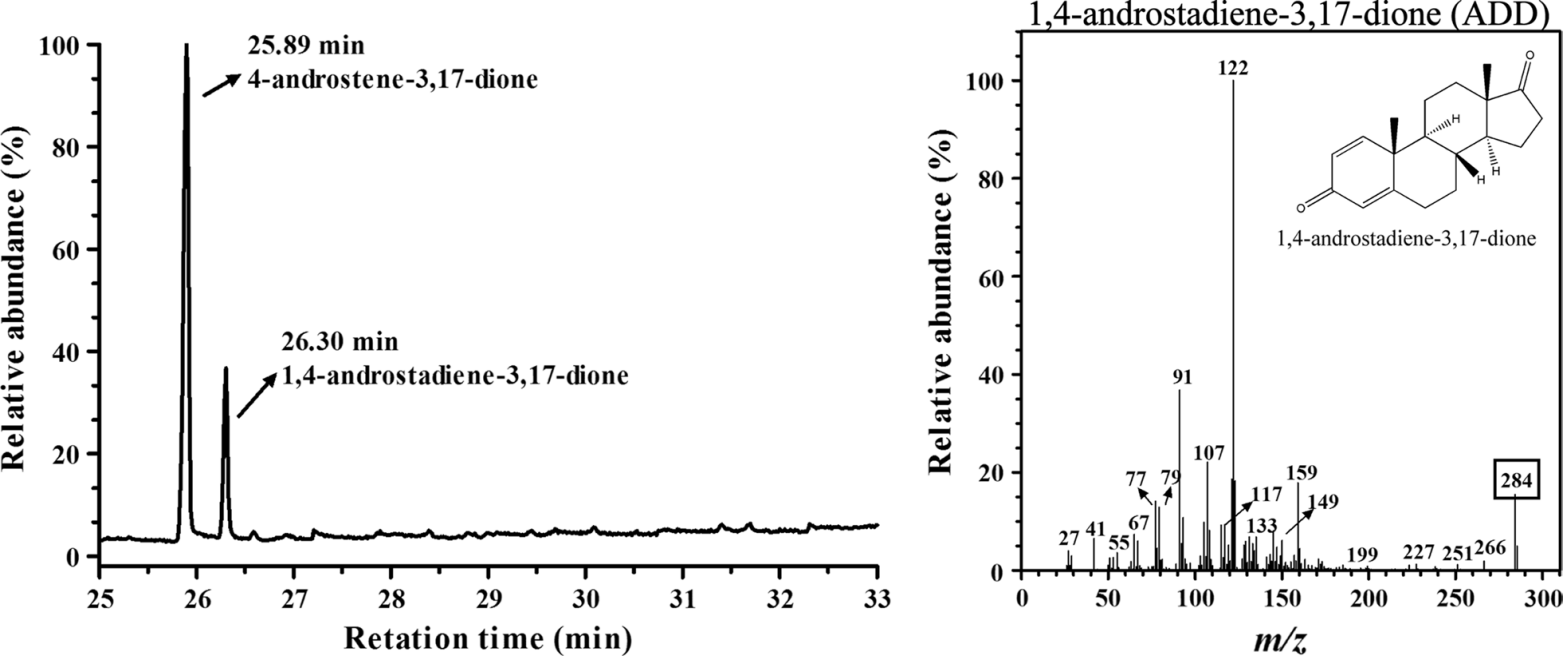
Product analysis of ADD by GC–MS

The Michaelis–Menten constant (*K*_m_) and the catalytic rate constant (*k*_cat_) of KsdD3 toward 3-ketosteroid substrates were determined to investigate the substrate selectivity. KsdD3 exhibited broad selectivity towards various 3-ketosteroid substrates (Table 1 and Additional file 1: Fig. S2). The highest enzyme activity was observed using testosterone as the substrate (74,648 nmol mg^−1^ min^−1^), followed by AD (59,811 nmol mg^−1^ min^−1^). The kinetic parameters showed that purified KsdD3 had the highest affinity (*K*_m_ = 42.05 μM) and the highest reaction rate (*k*_*cat*_ = 79.50 s^−1^) towards testosterone. The *k*_cat_/*K*_m_ of WT for testosterone (1.89 × 10^6^ M^−1^ s^−1^) was the highest in the tested substrates, which implied that KsdD3 possessed strong catalytic activity towards steroidal substrates with a C17 hydroxyl or carbonyl side chain compared with substrates with a longer side chain (i.e. cortisone acetate). AD was selected as the substrate for rational design because it has been used as the general substrate in activity assay in the previous research [17, 34]. The products 1,4-androstadiene-3,17-dione, boldenone, methandienone, prednisone, prednisone acetate, 1-dehydroprogesterone were characterized by GC–MS (Fig. 3 and Additional file 1: Fig. S3) and 16α,17α-epoxypregna-11α-hydroxy-1,4-diene-3,20-dione, 3-Hydroxy-9,10-secoandrosta-1,3,5(10)-triene-9,17-dione and 17α-hydroxypregna-1,4-diene-3,11,20-trione were identified using NMR (Additional file 1: Fig. S4).

**Table 1:** Kinetic parameters of KsdD3 WT and mutants toward nine substrates

Substrates	Mutants	*K*_m_ (µM)	*k*_*cat*_ (s^−1^)	*k*_*cat*_/*K*_m_ (× 10^6^ M^−1^ s^−1^)	Specific activity (nmol mg^−1^ min^−1^)	Relative activity (%)
AD	WT	47.43 ± 1.9	61.25 ± 2.1	1.29	59811 ± 823	100.00 ± 2.5
	W299A	34.89 ± 2.5	114.33 ± 5.1	3.28	112588 ± 1021	188.24 ± 3.8
	W299G	37.01 ± 1.8	101.24 ± 0.9	2.74	97618 ± 689	163.21 ± 3.2
Testosterone	WT	42.05 ± 4.3	79.50 ± 5.4	1.89	74648 ± 1427	124.81 ± 7.6
	W299A	33.25 ± 4.2	122.91 ± 3.9	3.70	118119 ± 1134	197.49 ± 4.2
	W299G	38.89 ± 2.9	92.88 ± 7.1	2.39	91506 ± 1341	152.99 ± 2.7
17-MT	WT	55.35 ± 3.1	56.94 ± 5.2	1.03	49929 ± 1538	83.48 ± 4.2
	W299A	44.63 ± 2.8	74.15 ± 1.8	1.66	69278 ± 838	115.83 ± 3.3
	W299G	52.18 ± 3.6	58.08 ± 2.7	1.11	54469 ± 790	91.07 ± 3.3
Cortisone	WT	50.04 ± 2.7	59.68 ± 4.7	1.19	56892 ± 899	95.12 ± 3.5
	W299A	42.85 ± 3.3	78.05 ± 9.2	1.82	73209 ± 1382	122.40 ± 2.9
	W299G	47.91 ± 2.1	60.73 ± 3.6	1.27	59446 ± 968	99.39 ± 3.4
CA	WT	95.08 ± 4.7	45.27 ± 3.5	0.48	31776 ± 740	53.13 ± 5.8
	W299A	75.93 ± 3.8	47.32 ± 1.5	0.62	37555 ± 1185	62.79 ± 3.5
	W299G	146.28 ± 5.6	39.18 ± 1.8	0.27	24294 ± 693	40.62 ± 2.3
11α-OH-P	WT	228.26 ± 7.1	19.20 ± 4.5	0.08	11660 ± 562	19.49 ± 3.5
	W299A	149.15 ± 4.6	33.59 ± 2.1	0.23	24041 ± 347	40.19 ± 6.4
	W299G	171.65 ± 3.9	26.05 ± 0.7	0.15	16799 ± 197	28.09 ± 4.2
9α-OH-AD	WT	145.11 ± 4.4	40.56 ± 4.6	0.28	24385 ± 361	40.77 ± 3.2
	W299A	68.49 ± 7.0	51.76 ± 3.3	0.76	41874 ± 712	70.01 ± 3.4
	W299G	107.03 ± 5.1	43.11 ± 5.4	0.40	30598 ± 294	51.16 ± 2.3
21-DC	WT	57.12 ± 2.8	55.18 ± 3.7	0.97	46677 ± 649	78.04 ± 2.5
	W299A	46.85 ± 0.9	62.06 ± 6.1	1.32	61375 ± 871	102.61 ± 5.2
	W299G	54.91 ± 3.6	57.71 ± 8.9	1.05	52702 ± 610	88.11 ± 3.9
Progesterone	WT	62.31 ± 4.8	53.47 ± 4.1	0.86	45022 ± 429	75.27 ± 2.9
	W299A	40.37 ± 3.7	87.12 ± 4.2	2.16	87199 ± 192	145.79 ± 3.8
	W299G	43.19 ± 2.8	74.82 ± 1.4	1.73	70217 ± 314	117.40 ± 2.2

### Structural analysis of the KsdD3 homology model

**Fig. 4:**
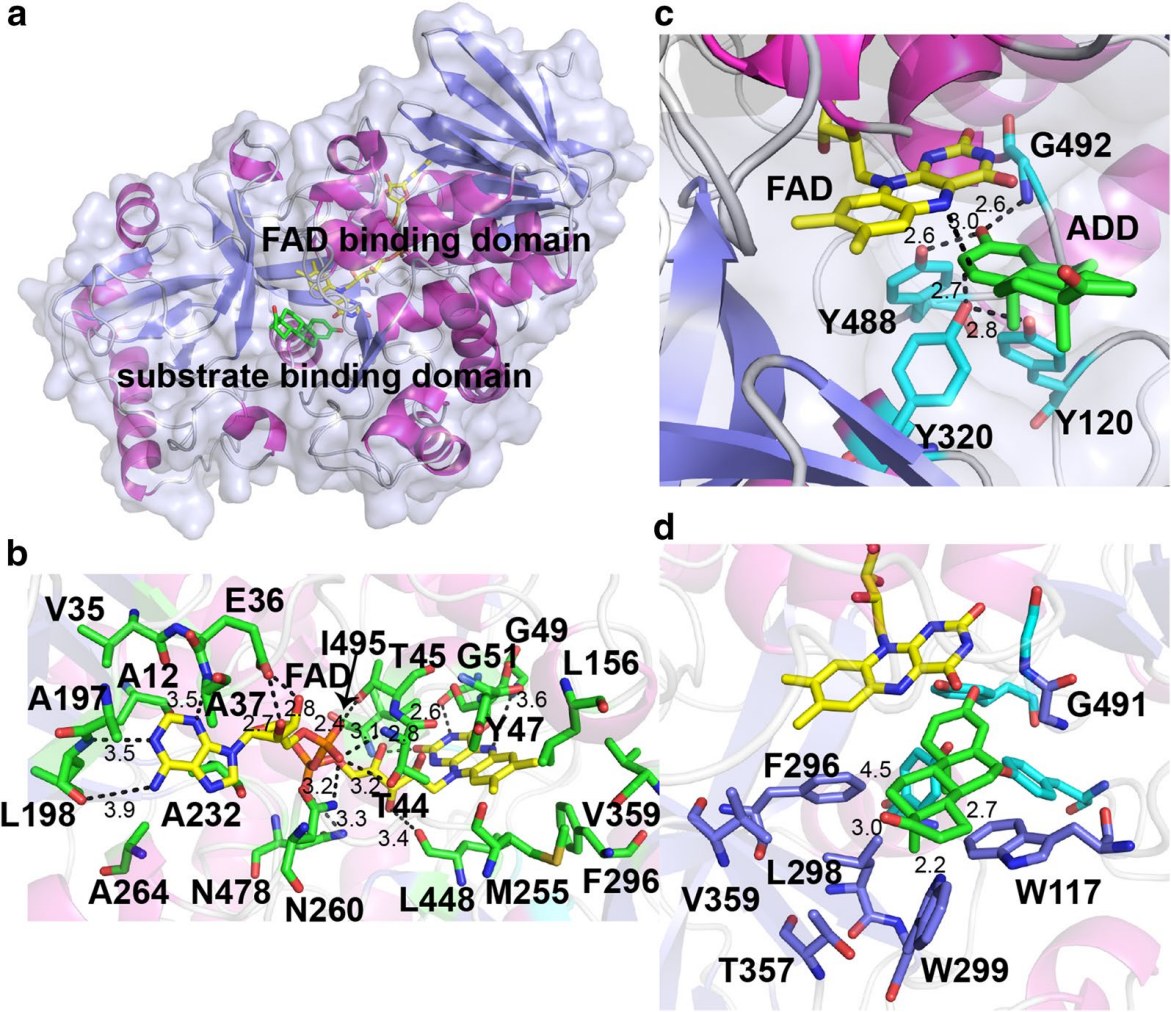
Structural analysis of the KsdD3-substrate complex model. **a** Ribbon representation of the KsdD3 overall structure. FAD and AD are shown as yellow and green stick, respectively. **b** The FAD-binding site. FAD was colored as yellow stick. **c** The active site of KsdD3. The catalytic residues are shown as cyan stick. **d** The predicted substrate binding model. The residues are shown as purple stick

A homology model of KsdD3 was generated using *R. erythropolis* SQ1 ∆1-KSTD1 as template (PDB ID: 4C3×, sequence identity 46%), which possessed a traditional Rossmann fold (nucleotide-binding fold) (Fig. 4a). KsdD3 contains a FAD binding domain and a catalytic domain. FAD is bound non-covalently to KsdD3 through a variety of interactions. The adenine moiety is located in a hydrophobic pocket formed mainly by the side chains of A12, V35, A37, A264, A197, L198 and A232. E36 has a bidentate hydrogen bonding interaction between the carboxylate group with the diol O2 and O3 atoms of ribose. The pyrophosphate oxygen atoms are hydrogen bonded to T44, T45, N260 and N478 of KsdD3. The isoalloxazine ring is surrounded by Y47, G49, G51, L156, M255, F296 and V359, which are almost all hydrophobic residues (Fig. 4b). The catalytic residues included Y120, Y320, Y488 and G492, which are conserved in the KsdD family (Fig. 4c). On the basis of the conserved catalytic mechanism of flavoenzymes [34], Y488 and G492 promote keto-enol tautomerization and increase the acidity of the C2 hydrogen atoms of the substrate. With the assistance of Y120, the general base Y320 abstracts the hydrogen from C2 as a proton, whereas FAD accepts the hydrogen from the C1 atom of the substrate as a hydride ion.

The substrate-binding domain shows a variety of different topologies in the KsdD family (Additional file 1: Fig. S5). The internal cavity is occupied by partially conserved hydrophobic residues: W117, F296, L298, W299, T357, V359 and G491 (Fig. 4d), which are predicted to interact with the buried substrate in this cavity.

### Comparison of KsdD3 with family enzymes

Sequence alignment shows that KsdD3 shares at most 46% sequence identity with *R. erythropolis* SQ1-KsdD1, which gives us only a traditional Rossmann fold (nucleotide-binding fold), a FAD binding domain and a catalytic domain. The mechanism of substrate recognition and selectivity are poor understood. Xie reported that S138 played an important role in maintaining the active center via hydrogen bond network of *Mycobacterium neoaurum* KsdD [43]. However, this residue is partial conserved in this family enzyme, which is H134 in *A. simplex* and S131 in *R. erythropolis* SQ1 (Additional file 1: Fig. S5). Shao found that V366S stabilized the active center and enhancing the interaction of AD in *M. neoaurum* KsdD [44]. V366 was positioned to D321 of *A. simplex* KsdD3. Both H134S and D321S in *A. simplex* KsdD3 abolished the catalytic activity, which implied that H134 and D321 placed the irreplaceable role in substrate recognition. E140 and Y472 of *M. neoaurum* KsdD were located at the same position as I136 and F423 in *A. simplex* KsdD3 [45]. They were reported to form a hydrophobic pocket near the active site, and were crucial for catalysis because I136E destroyed the activity toward AD (Additional file 1: Fig. S6). Two mutants of S325F and T503I from *R. erythropolis* SQ1-KsdD2 have also been proven to play important role in substrate recognition or catalytic reaction [46]. T503 (T450 in *A. simplex* KsdD3) are conserved while S325 (A281 in *A. simplex* KsdD3) are partial conserved. However, both residues were located far away from substrate-binding site, and were not considered to react with substrate.

The structural model of KsdD3 showed that F296, L298 and W299 are positioned at the entrance of substrate-binding cavity, and W117 is located at the inner cavity of substrate-binding cavity. Neither Trp is conserved in KsdD family. Furthermore, W299 and W117 form hydrophobic recognition wall between indolyl group and AD ring. This interaction relieves the steric interference between substrate and KsdD3. Hence, W299 and W117 were selected as the mutation sites for rational design.

### Structure-based rational design of KsdD3

**Fig. 5:**
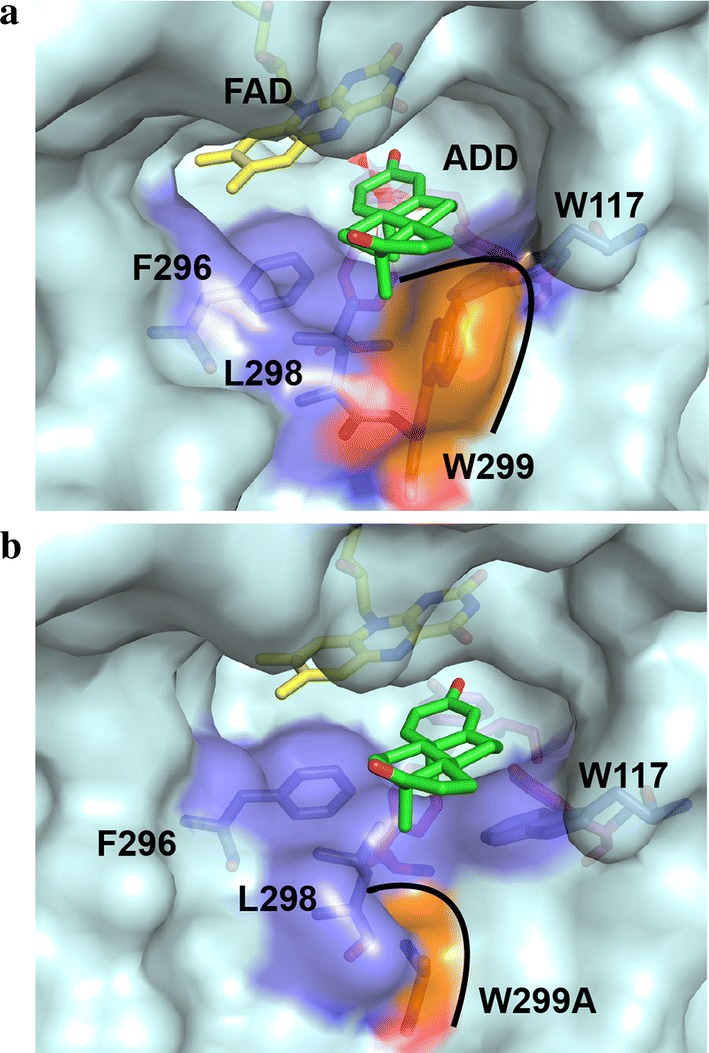
The structural model of substrate entrance of KsdD3 WT and W299A mutant with AD bound

A single point mutagenesis group containing 20 single mutants at 11 residues discussed above was constructed and evaluated to identify the role of specific amino acids in substrate selectivity and structure–function relationships. The mutation of residues Y115, W117, H134, I136, F296, L298 D321 and M361 inactivated the enzyme towards AD (Additional file 1: Fig. S6), indicating that these residues play a crucial role in substrate recognition. P139A/D showed a similar catalytic level as WT KsdD3. Interestingly, W299A mutant improved the catalytic activity toward AD, while W117 mutants inactivated KsdD3. The W299A mutant enlarged the substrate-binding cavity and relieved the steric interference with substrates, facilitating recognition of C17 hydroxyl/carbonyl steroidal substrates (Fig. 5). Therefore, the rational design was performed beginning with site-directed saturation mutagenesis of W299 to investigate its catalytic activity under guidance of structural model of KsdD3 homologue.

The results revealed that substitution by a few amino acids, i.e. Ala, Glu, Gly, His, Asn, Gln, Ser and Tyr, resulted in a higher activity towards AD than was shown by WT KsdD3 (Additional file 1: Fig. S7). W299D/K/M/R showed a similar catalytic activity as the WT enzyme. However, substitution with Cys, Phe, Ile, Leu, Pro or Val yielded a low activity. The biochemical data may imply that hydrophilic or hydrophobic residues with a smaller side chain (Ala or Gly) play a crucial role in substrate recognition at this position. W299A and W299G showed higher catalytic efficiency (*k*_cat_/*K*_m_) and specific activity towards the selected substrate, i.e. testosterone, AD and cortisone, compared with the WT enzyme (Table 1 and Additional file 1: Figs. S8 and S9).

Additional file 1: Fig. S10 shows the PMF profiles of KsdD3 WT and mutants dissociating along the substrate channel. The analysis revealed that the values of *∆G*_*off*_ are − 6.14 kcal/mol for W199A mutant and − 7.10 kcal/mol for W299G, which is lower than that of WT (− 11.45 kcal/mol) (Table 2). Therefore, as for the two mutants W299A and W299G, the association of substrate and the dissociation of product are easier than for WT. This is in agreement with the experimental values of the substrates (Table 1).

**Table 2:** Potential energy barriers of getting in the substrate for the wild-type and mutants

system	∆G_off_ (kcal/mol)	∆∆G_off_ (kcal/mol)
WT	− 11.45	–
W299A	− 6.14	5.31
W299G	− 7.10	4.35

### Bioconversion of steroids by KsdD3 enzyme and resting cells

**Fig. 6:**
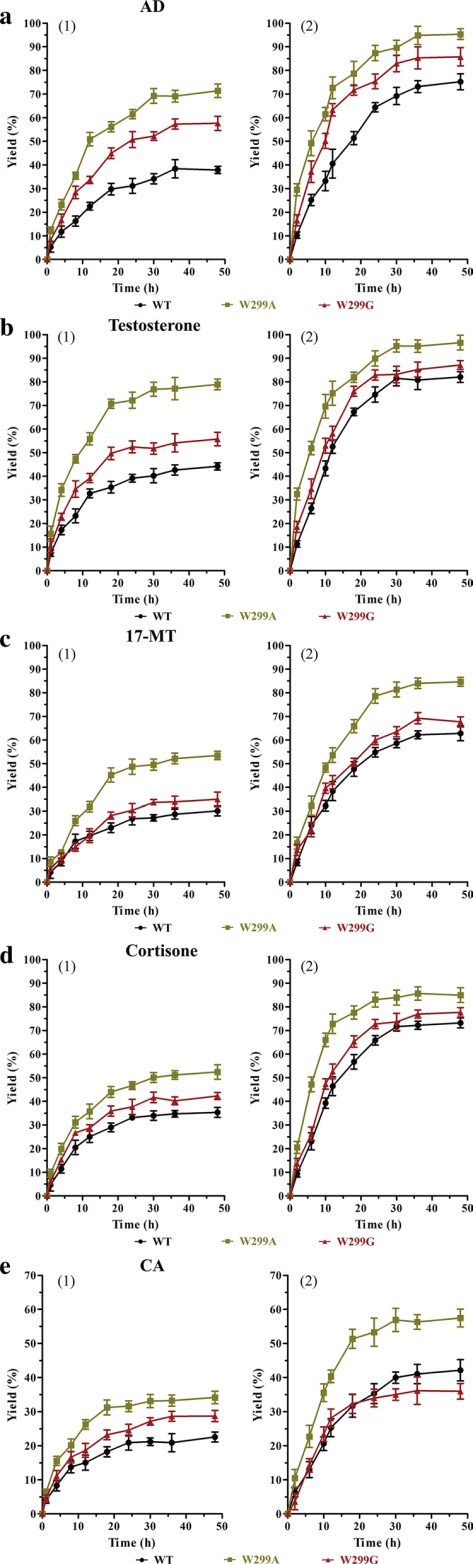
The bioconversion of AD (**a**), testosterone (**b**), 17-MT (**c**), cortisone (**d**) and CA (**e**) by purified KsdD3 enzyme (1) and *E. coli* resting cells (2)

The bioconversion of steroidal substrates with purified KsdD3 enzyme and *E. coli* resting cells (Fig. 6) over 48 h was investigated. The ADD productivity gradually increased, and reached a plateau at 30 h. WT, W299A and W299G showed maximum ADD yields of 38, 71 and 58%, respectively, catalyzed by the KsdD3 enzyme at 48 h.

The resting cells of recombinant *E. coli* BL21(DE3) harbouring KsdD3 WT, W299A and W299G showed ADD yields of 75, 95 and 86%, respectively. The yields of other steroidal substrates (testosterone, 17-MT, cortisone, CA) were about 18–72% by KsdD3 enzymes and 75–95% by resting cells. W299A showed the highest product yields (testosterone to boldenone) of 79% by enzymatic catalysis, and 97% by resting cells, respectively. The boldenone productivities were 0.047 and 0.052 g L^−1^ h^−1^, respectively (Additional file 1: Table S3).

## Conclusions

In summary, we have characterized KsdD3, showing that this enzyme exhibits organic solvent tolerance. Residues related to substrate recognition around the active site were investigated and mutation on W299 improved the substrate selectivity and activity based on rational design and protein engineering technologies. The SMD results and PMF calculations show that KsdD3 mutants need to overcome a lower potential energy barrier than the WT. Furthermore, both the KsdD3 enzyme mutants and *E. coli* strains harbouring KsdD3 genes improved the yields observed with several steroidal substrates.

## Additional file

**Additional file 1: Table S1.** Primers used for the construction of KsdD3 WT and mutants. **Table S2.** Energy minimization of KsdD3 with and without substrate for the wild-type and mutants. **Table S3.** Productivity of steroid bioconversion in 48 h (g L^−1^ h^−1^). **Fig. S1.** Purification of KsdD3. **Fig. S2.** The relative activity of KsdD3 mutants towards steroidal substrates. **Fig. S3.** Products analysis of steroidal substrates by GC–MS. **Fig. S4. A** Spectral data for hydrocarbons analysis of 16α,17α-epoxypregna-11α-hydroxy-1,4-diene-3,20-dione by NMR. **B** Spectral data for hydrocarbons analysis of 3-hydroxy-9,10-secoandrosta-1,3,5(10)-triene-9,17-dione by NMR. **C** Spectral data for hydrocarbons analysis of 17α-hydroxypregna-1,4-diene-3,11,20-trione by NMR. **Fig.** S5. Amino acid sequence alignments of 3-ketosteroid-∆1-dehydrogenase from different strains. **Fig. S6.** The relative catalytic activity of KsdD3 mutants toward AD. **Fig. S7.** The relative catalytic activity of saturation mutagenesis on W299 toward AD. **Fig. S8.** The relative catalytic activity of KsdD WT and mutants toward various steroidal substrates. **Fig. S9.** Michaelis–Menten plots of KsdD3 WT and W299A, W299G mutants toward nine steroidal substrates. **Fig. S10.** Potential of mean force (PMF) profiles of the KsdD3 wild type and mutants over the distance along the substrate channel.

## Abbreviations

AD: 4-androstene-3,17-dione
ADD: androst-1,4-diene-3,17-dione
17-MT: methyltestosterone
CA: cortisone acetate
PA: prednisone acetate
11α-OH-P: 11α-hydroxy-16,17α-epoxyprogesterone
9α-OH-AD: 9α-hydroxy-4-androstene-3,17-dione
21-DC: 21-deoxycortisone
IPTG: isopropy-β-d-thiogalactoside
DCPIP: 2,6-dichlorophenolindophenol
PMS: phenazine methosulphate
FAD: flavin adenine dinucleotide
SMD: steered molecular dynamics
