# Supplementary material for: Engineering of 3-ketosteroid-∆1-dehydrogenase based site-directed saturation mutagenesis for efficient biotransformation of steroidal substrates
Source: Microb Cell Fact. 2018 Sep 10;17:141. doi: 10.1186/s12934-018-0981-0 (PMC6130075; doi:10.1186/s12934-018-0981-0)
Supplement: Supplementary file 1 — Additional file 1: Table S1. Primers used for the construction of KsdD3 WT and mutants. Table S2. Energy minimization of KsdD3 with and without substrate for the wild-type and mutants. Table S3. Productivity of steroid bioconversion in 48 h (g L−1 h−1). Fig. S1. Purification of KsdD3. Fig. S2. The relative activity of KsdD3 mutants towards steroidal substrates. Fig. S3. Products analysis of steroidal substrates by GC–MS. Fig. S4. A Spectral data for hydrocarbons analysis of 16α,17α-epoxypregna-11α-hydroxy-1,4-diene-3,20-dione by NMR. B Spectral data for hydrocarbons analysis of 3-hydroxy-9,10-secoandrosta-1,3,5(10)-triene-9,17-dione by NMR. C Spectral data for hydrocarbons analysis of 17α-hydroxypregna-1,4-diene-3,11,20-trione by NMR. Fig. S5. Amino acid sequence alignments of 3-ketosteroid-∆1-dehydrogenase from different strains. Fig. S6. The relative catalytic activity of KsdD3 mutants toward AD. Fig. S7. The relative catalytic activity of saturation mutagenesis on W299 toward AD. Fig. S8. The relative catalytic activity of KsdD WT and mutants toward various steroidal substrates. Fig. S9. Michaelis–Menten plots of KsdD3 WT and W299A, W299G mutants toward nine steroidal substrates. Fig. S10. Potential of mean force (PMF) profiles of the KsdD3 wild type and mutants over the distance along the substrate channel. [file 12934_2018_981_MOESM1_ESM.docx]

**Additional Material**

**Engineering of 3-ketosteroid-Δ^1^-dehydrogenase based site-directed saturation mutagenesis for efficient biotransformation of steroidal substrates**

Shuhong Mao^1,2,3,4‡^, Jian-Wen Wang^4‡^, Fufeng Liu^1,2,3,4‡^, Zhangliang Zhu^4^, Dengke Gao^4^, Qianqian Guo^4^, Panpan Xu^4^, Zheng Ma^4^, Yali Hou^4^, Xiaotao Cheng^4^, Dengyue Sun^4^, Fuping Lu^1,2,3,4,5^* and Hui-Min Qin^1,2,3,4,5^*

^1^ State Key Laboratory of Food Nutrition and Safety;

^2^ Key Laboratory of Industrial Fermentation Microbiology, Ministry of Education;

^3^ Tianjin Key Laboratory of Industrial Microbiology;

^4^ College of Biotechnology, Tianjin University of Science and Technology;

^5^ National Engineering Laboratory for Industrial Enzymes, Tianjin 300457, People’s Republic of China

^‡^These authors contributed equally to this work.

*Corresponding authors: F. Lu: lfp@tust.edu.cn; H.-M. Qin: huiminqin@tust.edu.cn

**Additional file**

**Table S1** Primers used for the construction of KsdD3 WT and mutants

**Table S2** Energy minimization of KsdD3 with and without substrate for the wild-type and mutants

**Table S3** Productivity of steroid bioconversion in 48 h (g•L^-1^•h^-1^)

**Fig. S1** Purification of KsdD3.

**Fig. S2** The relative activity of KsdD3 mutants towards steroidal substrates.

**Fig. S3** Production analysis by GC-MS

**Fig. S4A** Spectral data for hydrocarbons analysis of 16α,17α-epoxypregna-11α-hydroxy-1,4-diene-3,20-dione by NMR

**Fig. S4B** Spectral data for hydrocarbons analysis of 3-hydroxy-9,10-secoandrosta-1,3,5(10)-triene-9,17-dione by NMR

**Fig. S4C** Spectral data for hydrocarbons analysis of 17α-hydroxypregna-1,4-diene-3,11,20-trione by NMR

**Fig. S5** Amino acid sequence alignments of 3-ketosteroid-Δ1-dehydrogenase from different strains

**Fig. S6** The relative catalytic activity of KsdD3 mutants toward AD

**Fig. S7** The relative catalytic activity of saturation mutagenesis on W299 toward AD

**Fig. S8** The relative catalytic activity of KsdD WT and mutants toward various steroidal substrates

**Fig. S9** Michaelis–Menten plots of KsdD3 WT and W299A, W299G mutants toward nine steroidal substrates

**Fig. S10** Potential of mean force (PMF) profiles of the KsdD3 wild type and mutants over the distance along the substrate channel

**Table S1 Primers used for the construction of KsdD3 WT and mutants**

| WT  Mutants | Primers | |
| --- | --- | --- |
|  | Forward | Reverse |
| KsdD | GGAATTC**CATATG**ATGGACTGGGCAGAG | CCG**GAATTC**TCATCGCGCGTCCTC |
| Y115A | **GC**CCCGTGGCCGGATTACTTTGG | ACTCTCGAAGCTGAAGTGGTCGTCTTC |
| Y115R | **CG**CCCGTGGCCGGATTACTTTGG | ACTCTCGAAGCTGAAGTGGTCGTCTTC |
| W117A | **GC**GCCGGATTACTTTGGCGATGC | CGGGTAACTCTCGAAGCTGAAGTGG |
| W117F | **TC**CCGGATTACTTTGGCGATGCACC | ACGGGTAACTCTCGAAGCTGAAGTGGTC |
| W117I | **ATA**CCGGATTACTTTGGCGATGC | CGGGTAACTCTCGAAGCTGAAGTG |
| W117Y | **AT**CCGGATTACTTTGGCGATGCACC | ACGGGTAACTCTCGAAGCTGAAGTGGTC |
| H134S | **AG**CATTATTCCGACCCCGCTGCC | GCGCTGGCCGTCACGAC |
| I136E | **GAG**CCGACCCCGCTGCCTGTT | AATGTGGCGCTGGCCGTC |
| I136N | **A**TCCGACCCCGCTGCCTG | TAATGTGGCGCTGGCCGTCAC |
| P139A | **G**CGCTGCCTGTTCCGAGCG | GGTCGGAATAATGTGGCGCTGG |
| P139D | **GAT**CTGCCTGTTCCGAGCGCAC | GGTCGGAATAATGTGGCGCTGG |
| P139E | **GA**GCTGCCTGTTCCGAGCGC | GGTCGGAATA ATGTGGCGCTG |
| P139S | **T**CGCTGCCTGTTCCGAGCG | GGTCGGAATAATGTGGCGCTGG |
| P155E | **GA**GCTGGATAATGATCGTTTAGGCACC | GCCACGAACCACTTCACGTAATTC |
| F296A | **GC**TGCCCTGTGGTTTACCGGCG | GGCGCTGCGACCGTCAG |
| F296G | **GG**TGCCCTGTGGTTTACCGGCG | GGCGCTGCGACCGTCAGG |
| L298V | **G**TGTGGTTTACCGGCGGTATCTTTG | GGCAAAGGCGCTGCGAC |
| D321S | **AG**TCGTCTGGGCCGCGCAG | ATACGGGGCGCTTTCATTCACG |
| M361N | **AT**GTGGACGAAGAACAGTATGTGGCCG | TGCTCACGTTGGTGGCACG |
| W299A | **GC**GTTTACCGGCGGTATCTTTGTGGACG | CAGGGCAAAGGCGCTGCG |
| W299C | **C**TTTACCGGCGGTATCTTTGTGGAC | CACAGGGCAAAGGCGCTGC |
| W299D | **GAT**TTTACCGGCGGTATCTTTGTGGAC | CAGGGCAAAGGCGCTGC |
| W299E | **GA**GTTTACCGGCGGTATCTTTGTGGAC | CAGGGCAAAGGCGCTGC |
| W299F | **TC**TTTACCGGCGGTATCTTTGTGGACG | ACAGGGCAAAGGCGCTGCG |
| W299G | **G**GGTTTACCGGCGGTATCTTTGTGGAC | CAGGGCAAAGGCGCTGCG |
| W299H | **CAT**TTTACCGGCGGTATCTTTGTGGACG | CAGGGCAAAGGCGCTGCG |
| W299I | **ATA**TTTACCGGCGGTATCTTTGTGGAC | CAGGGCAAAGGCGCTGC |
| W299K | **AA**GTTTACCGGCGGTATCTTTGTGGAC | CAGGGCAAAGGCGCTGC |
| W299L | **T**GTTTACCGGCGGTATCTTTGTGGAC | ACAGGGCAAAGGCGCTGC |
| W299M | **AT**GTTTACCGGCGGTATCTTTGTGGAC | CAGGGCAAAGGCGCTGC |
| W299N | **AAT**TTTACCGGCGGTATCTTTGTGGAC | CAGGGCAAAGGCGCTGC |
| W299P | **CC**GTTTACCGGCGGTATCTTTGTGGAC | CAGGGCAAAGGCGCTGCG |
| W299Q | **CA**GTTTACCGGCGGTATCTTTGTGGACG | CAGGGCAAAGGCGCTGCG |
| W299R | **A**GGTTTACCGGCGGTATCTTTGTGGAC | CAGGGCAAAGGCGCTGCG |
| W299S | **C**GTTTACCGGCGGTATCTTTGTGGAC | ACAGGGCAAAGGCGCTGC |
| W299T | **AC**GTTTACCGGCGGTATCTTTGTGGACG | CAGGGCAAAGGCGCTGCG |
| W299V | **GT**GTTTACCGGCGGTATCTTTGTGGAC | CAGGGCAAAG GCGCTGC |
| W299Y | **AT**TTTACCGGCGGTATCTTTGTGGACG | ACAGGGCAAAGGCGCTGC |

**Table S2 Energy minimization of KsdD3 with and without substrate for the wild-type and mutants**

| system | E_min_ without AD  (kJ/mol) | E_min_ with AD (kJ/mol) |
| --- | --- | --- |
| WT | -730361 | -780365 |
| W299A | -889872 | -900902 |
| W299G | -903028 | -921104 |

**Table S3 Productivity of steroid bioconversion in 48 h (g·L-1·h-1)**

|  |  | AD | TS | 17-MT | Cortisone | CA |
| --- | --- | --- | --- | --- | --- | --- |
| KsdD3 | WT | 0.023±0.002 | 0.026±0.003 | 0.018±0.004 | 0.021±0.004 | 0.013±0.001 |
|  | W299A | 0.043±0.001 | 0.047±0.001 | 0.032±0.002 | 0.031±0.003 | 0.020±0.006 |
|  | W299G | 0.034±0.005 | 0.033±0.002 | 0.021±0.003 | 0.025±0.002 | 0.017±0.002 |
|  | | | | | | |
| *E. coli* | WT | 0.039±0.001 | 0.044±0.003 | 0.034±0.002 | 0.045±0.004 | 0.023±0.003 |
|  | W299A | 0.050±0.003 | 0.052±0.002 | 0.046±0.004 | 0.047±0.003 | 0.031±0.002 |
|  | W299G | 0.046±0.002 | 0.049±0.001 | 0.037±0.002 | 0.042±0.001 | 0.020±0.001 |
|  | | | | | | |


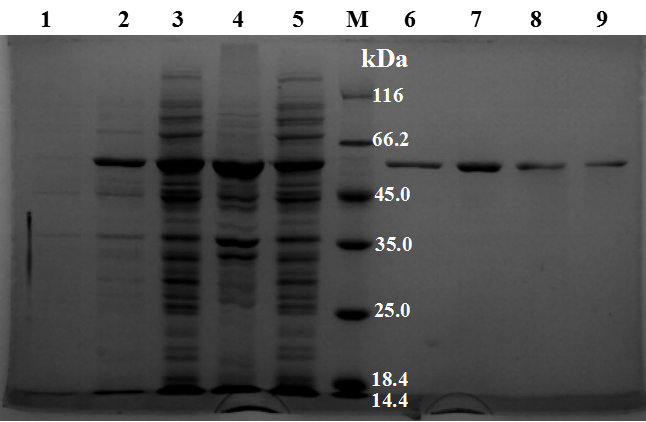


**Fig. S1** Purification of KsdD3. Lane 1: before induction; Lane 2: after induction; Lane 3: supernatant; Lane 4: precipitant; Lane 5: flowthrough; Lanes 6-9: elution.

**Fig. S2** The relative activity of KsdD3 mutants towards steroidal substrates. The activity of KsdD3 WT toward AD is represented as 100 and the error bars are standard deviations (n = 3).
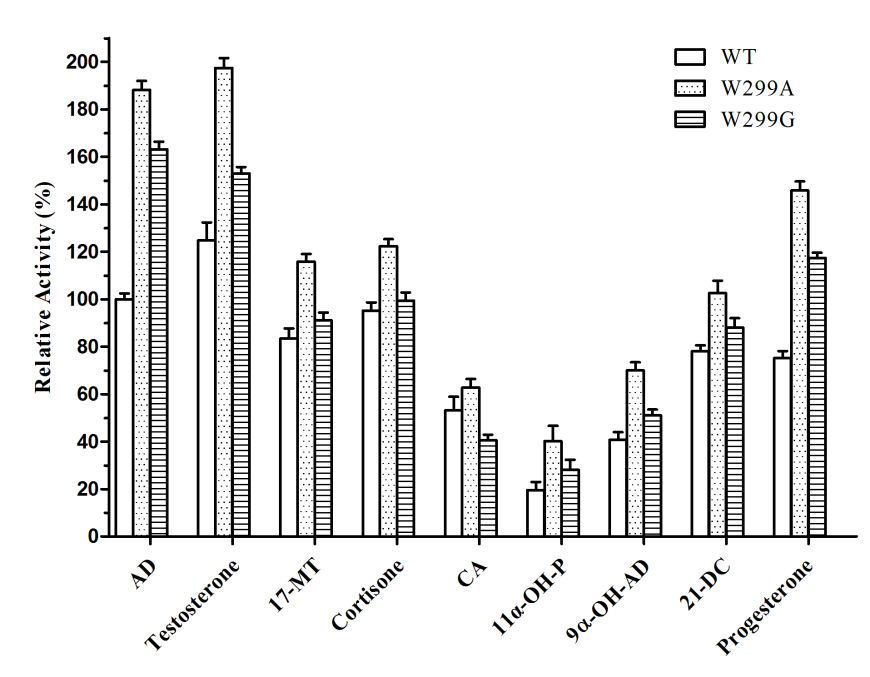


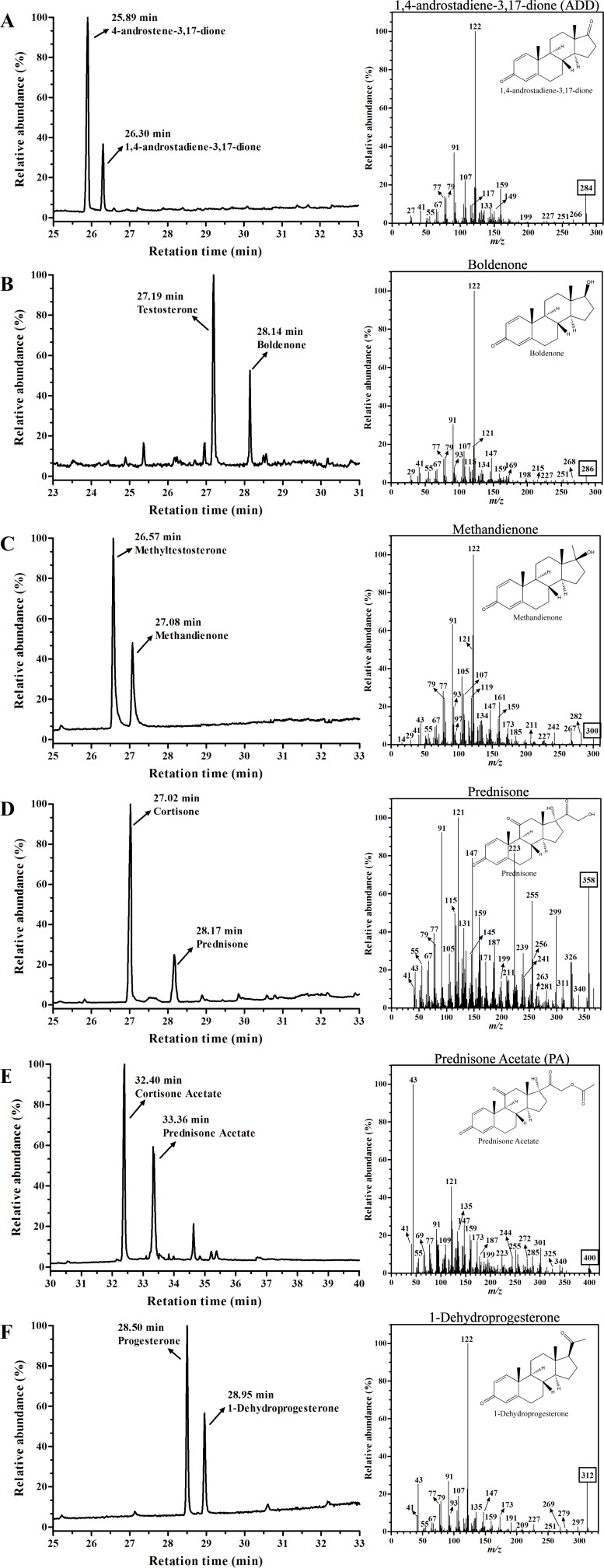


**Fig. S3 Production analysis by GC-MS**


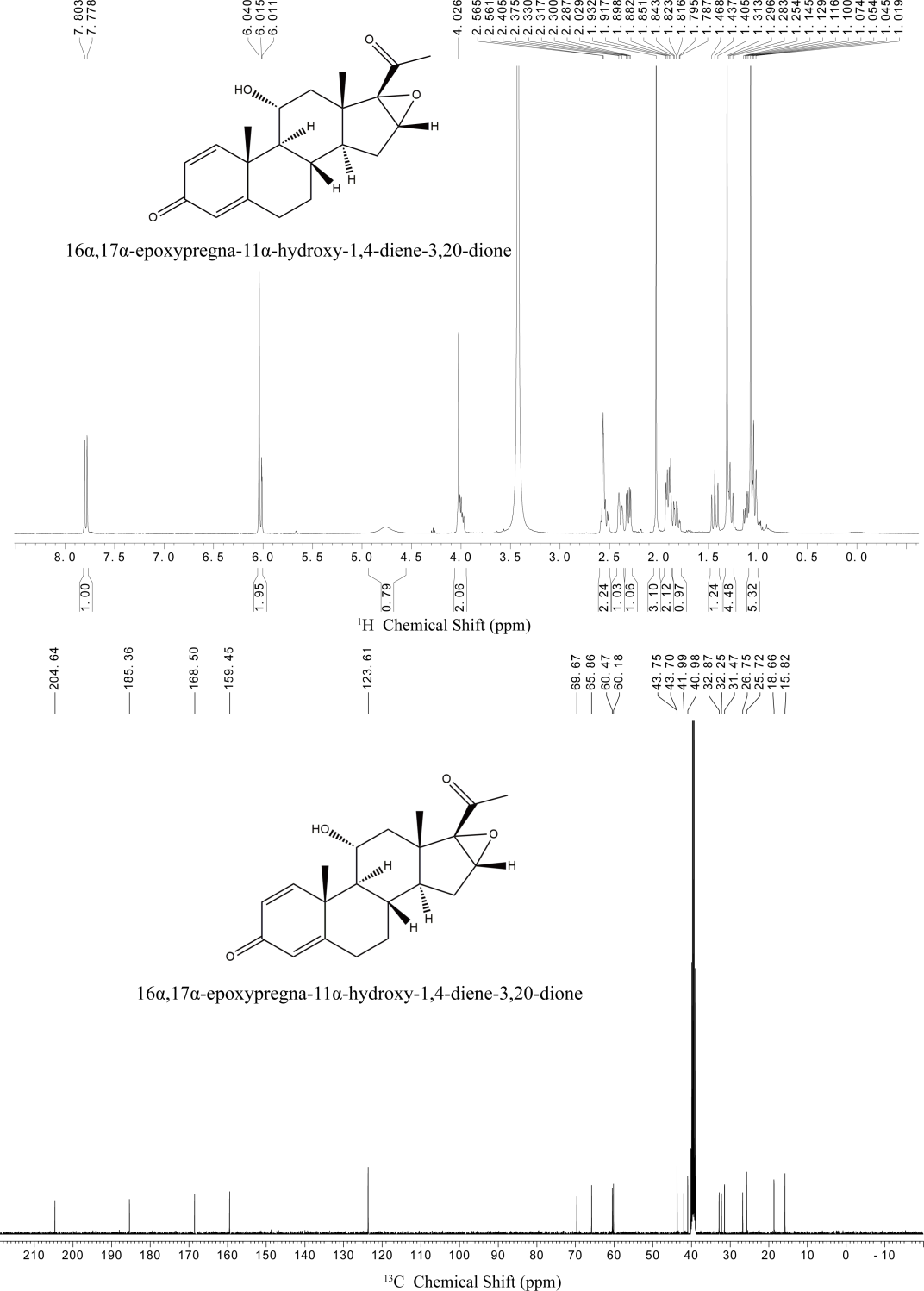


**Fig. 4A Spectral data for hydrocarbons analysis of 16α,17α-epoxypregna-11α-hydroxy-1,4-diene-3,20-dione by NMR.**

^1^H NMR (400 MHz, DMSO): δ 7.79 (d, *J* = 10.0Hz, 1H), 6.12 – 5.93 (m, 2H), 4.76 (s,1H),4.06 – 3.91 (m, 2H), 2.60 – 2.48 (m, 2H), 2.43 – 2.28 (m, 2H), 2.03 (s, 3H), 1.91 (dd, *J* = 13.6, 6.2 Hz, 2H), 1.83 (dd, *J* = 11.2, 3.0 Hz, 1H), 1.49 – 1.38 (t, *J* = 12.6Hz, 1H), 1.34 – 1.23 (m, 4H), 1.09 – 1.00 (m, 5H); ^13^C NMR (100 MHz, DMSO): δ 204.64, 185.36, 168.50, 159.45, 123.61, 69.67, 65.86, 60.47, 60.18, 43.75, 43.70, 41.99, 40.98, 32.87, 32.25, 31.47, 26.75, 25.72, 18.66, 15.82.


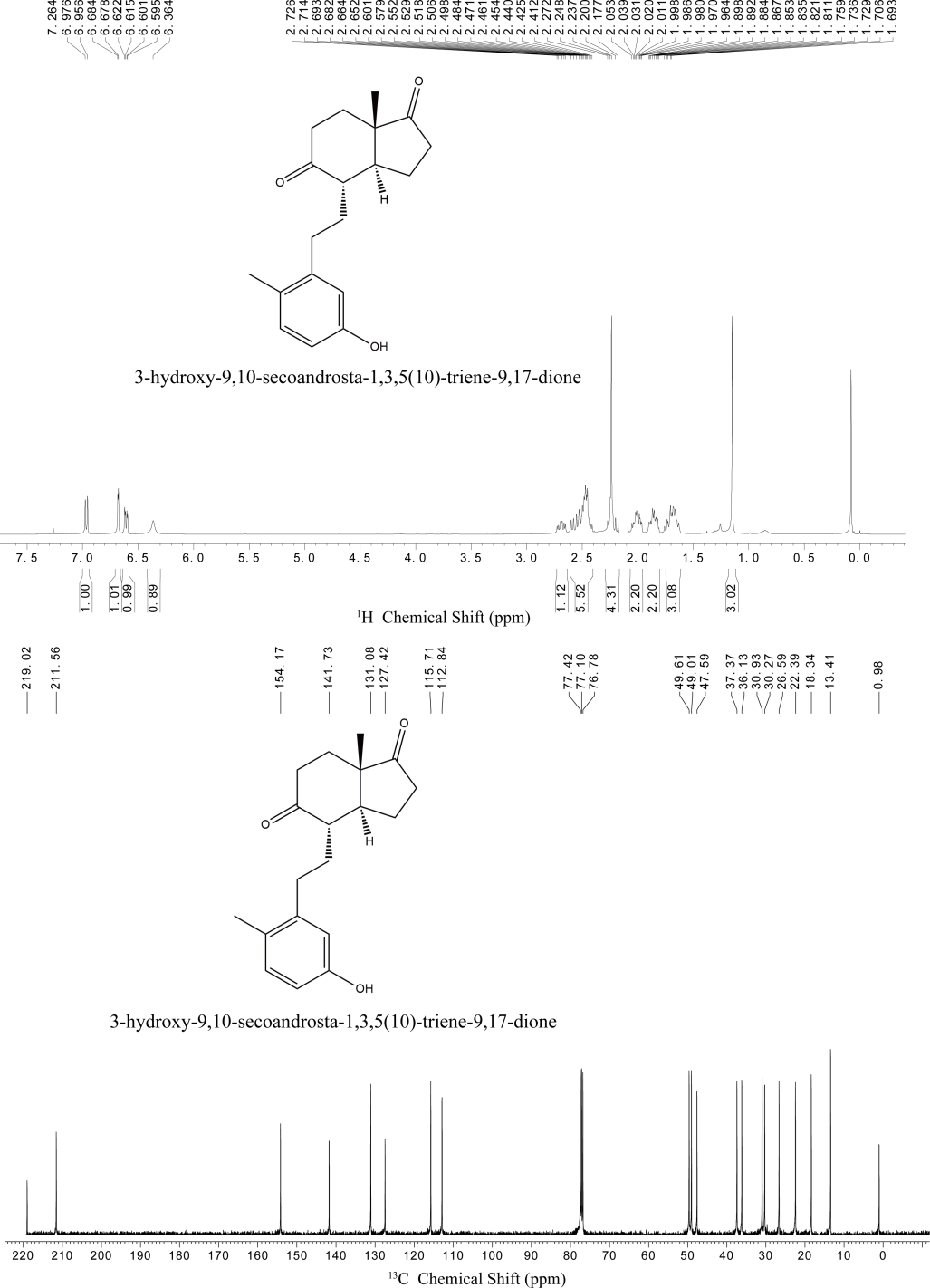


**Fig. 4B Spectral data for hydrocarbons analysis of 3-hydroxy-9,10-secoandrosta-1,3,5(10)-triene-9,17-dione by NMR.**

^1^H NMR (400 MHz, CDCl_3_): δ 6.97 (d, *J* = 8.2 Hz, 1H), 6.68 (d, *J* = 2.5 Hz, 1H), 6.61 (dd, *J* = 8.1, 2.5 Hz, 1H), 6.36 (s, 1H), 2.75 – 2.63 (m, 1H), 2.62 – 2.38 (m, 5H), 2.31 – 2.16 (m, 4H), 2.11 – 1.95 (m, 2H), 1.93 – 1.80 (m, 2H), 1.77 – 1.57 (m, 3H), 1.15 (s, 3H); ^13^C NMR (100 MHz, CDCl3): δ 219.02, 211.56, 154.17, 141.73, 131.08, 127.42, 115.71, 112.84, 77.42, 77.10, 76.78, 49.61, 49.01, 47.59, 37.37, 36.13, 30.93, 30.27, 26.59, 22.39, 18.34, 13.41, 0.98.


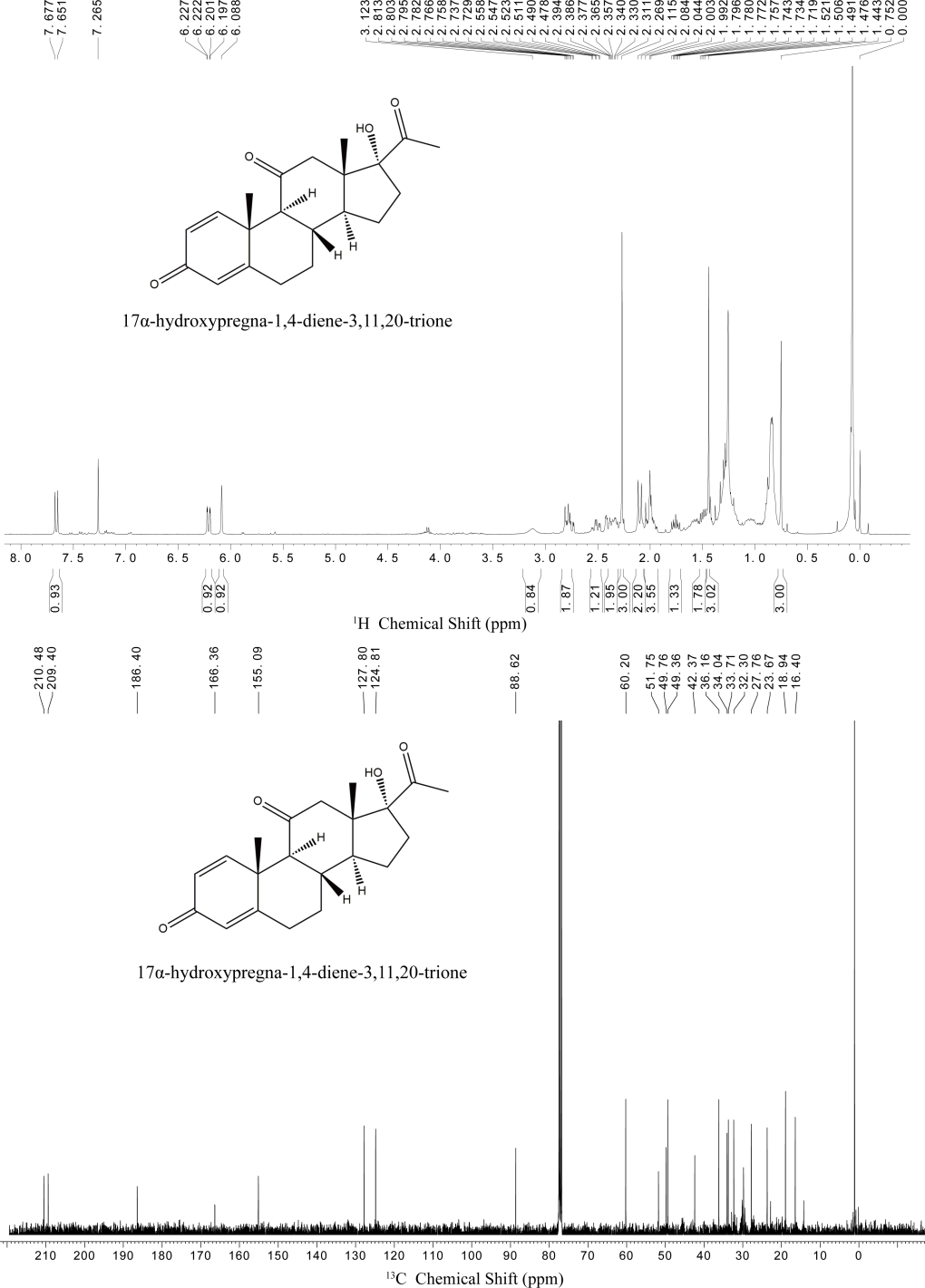


**Fig. 4C Spectral data for hydrocarbons analysis of 17α-hydroxypregna-1,4-diene-3,11,20-trione by NMR.**

^1^H NMR (400 MHz, CDCl_3_): δ 7.66 (d, *J* = 10.2 Hz, 1H), 6.21 (dd, *J* = 10.2, 1.8 Hz, 1H), 6.09 (s, 1H), 3.12 (s, 1H), 2.85 – 2.75 (m, 2H), 2.52 (td, *J* = 13.5, 4.2 Hz, 1H), 2.45 – 2.31 (m, 3H), 2.27 (s, 3H), 2.10 (d, *J* = 12.3 Hz, 2H), 2.05 – 1.92 (m, 3H), 1.76 (m, 2H), 1.50 (dd, *J* = 12.1, 6.2 Hz, 3H), 1.44 (s, 3H); ^13^C NMR (100 MHz, CDCl3): δ 210.48, 209.40, 186.40, 166.36, 155.09, 127.80, 124.81, 88.62, 60.20, 51.75, 49.76, 49.36, 42.37, 36.16, 34.04, 33.71, 32.30, 27.76, 23.67, 18.94, 16.40.


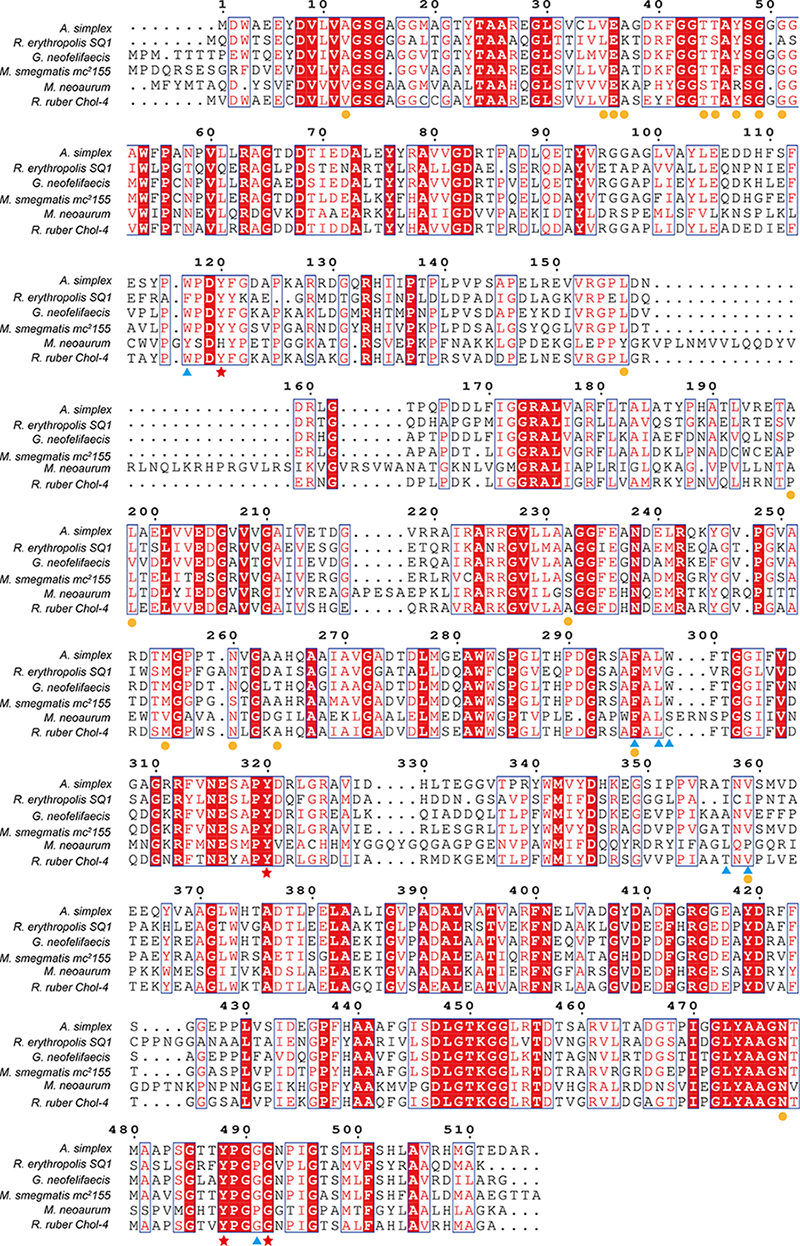


**Fig. S5 Amino acid sequence alignments of 3-ketosteroid-Δ^1^-dehydrogenase from different strains.** The catalytic residues were marked as red asterisk. FAD binding residues were marked as yellow circle. The substrate-binding residues were marked as blue triangle.

*A. simplex*: *Arthrobacter simplex*, AIY19527.1; *R. erythropolis* SQ1: *Rhodococcus erythropolis* SQ1, AF096929.1; *G. neofelifaecis*: *Gordonia neofelifaecis*, WP_009680993.1; *M. smegmatis* mc^2^155: *Mycobacterium smegmatis* mc^2^155, YP887187; *M. neoaurum*: *Mycobacterium neoaurum* ATCC 25795, GQ411074.1; *R. ruber* Chol-4: *Rhodococcus ruber* Chol-4, AFH57399.1.


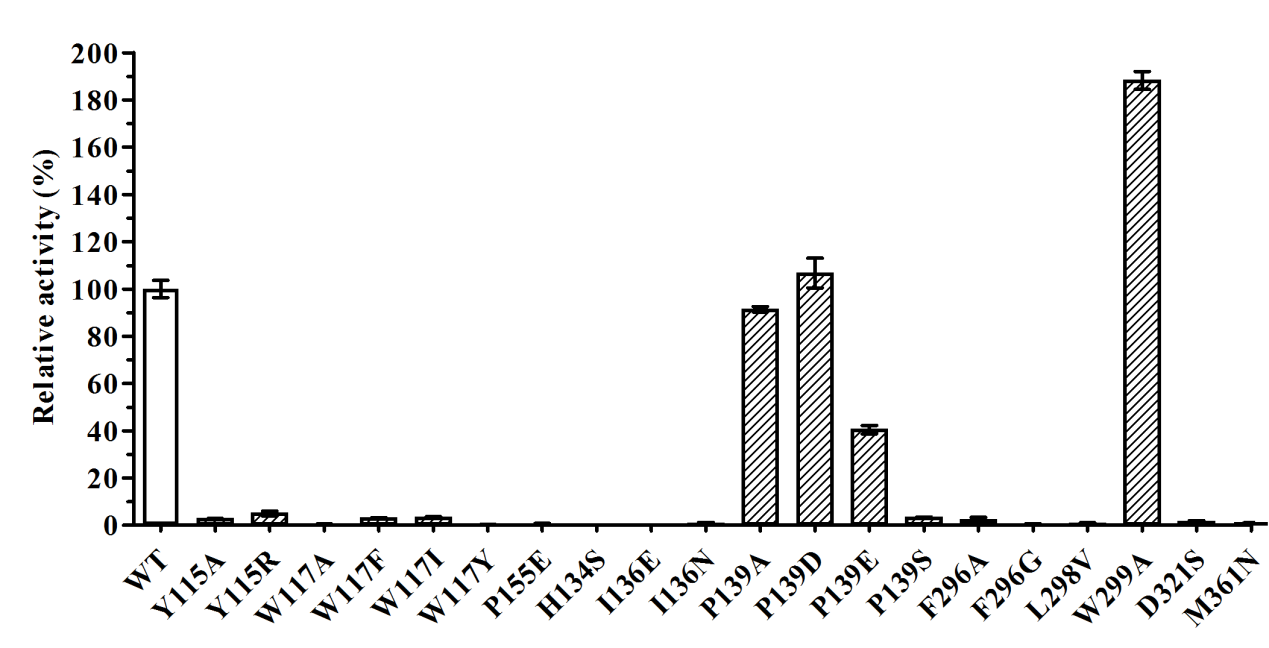


**Fig. S6 The relative catalytic activity of KsdD3 mutants toward AD**





**Fig. S7 The relative catalytic activity of saturation mutagenesis on W299 toward AD**





**Fig. S8 The relative catalytic activity of KsdD WT and mutants toward various steroidal substrates.** The activities of wild-type KsdD3 are represented as 100, respectively and the error bars are standard deviations (n =3).


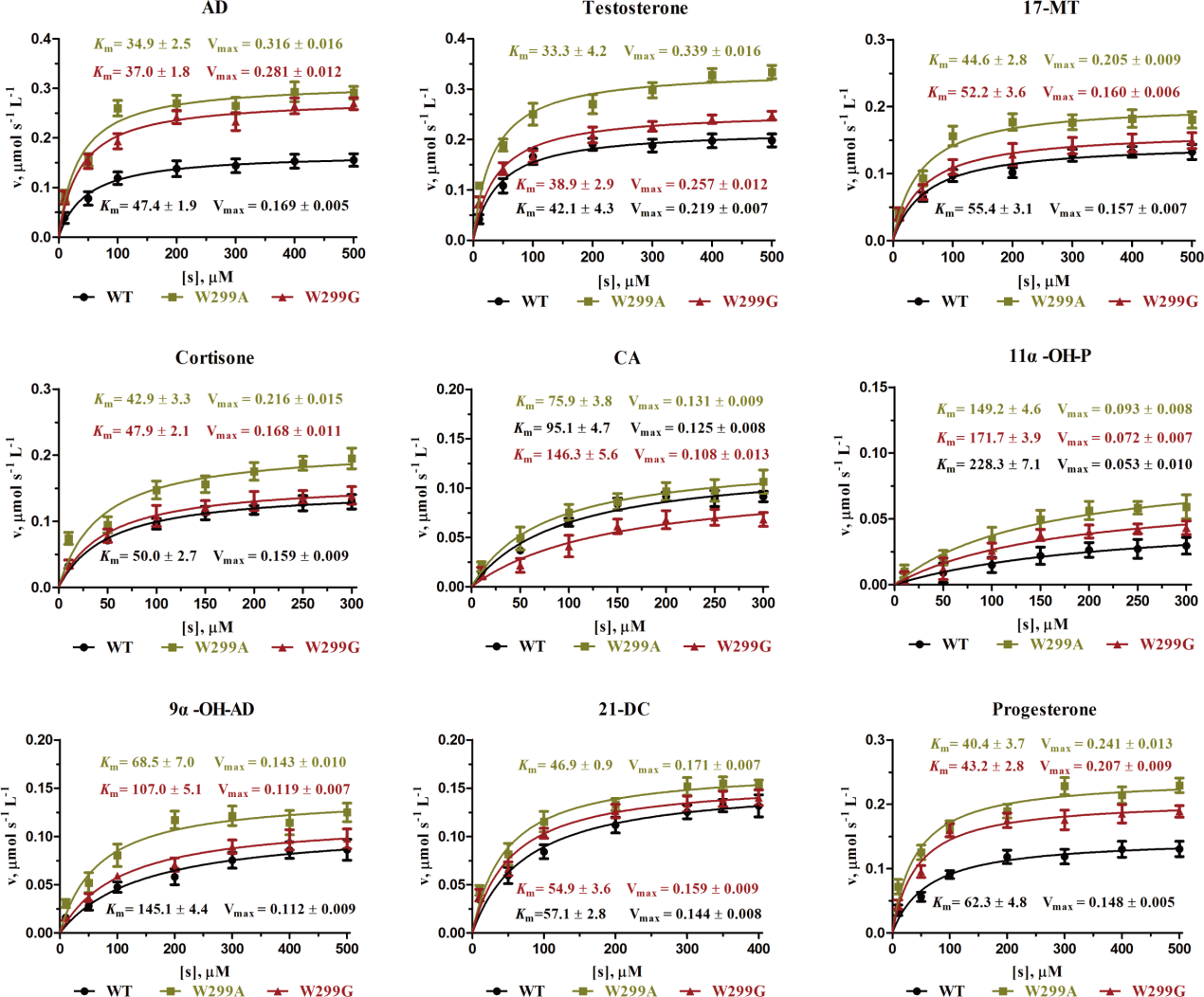


**Fig. S9 Michaelis–Menten plots of KsdD3 WT and W299A, W299G mutants toward nine steroidal substrates.**


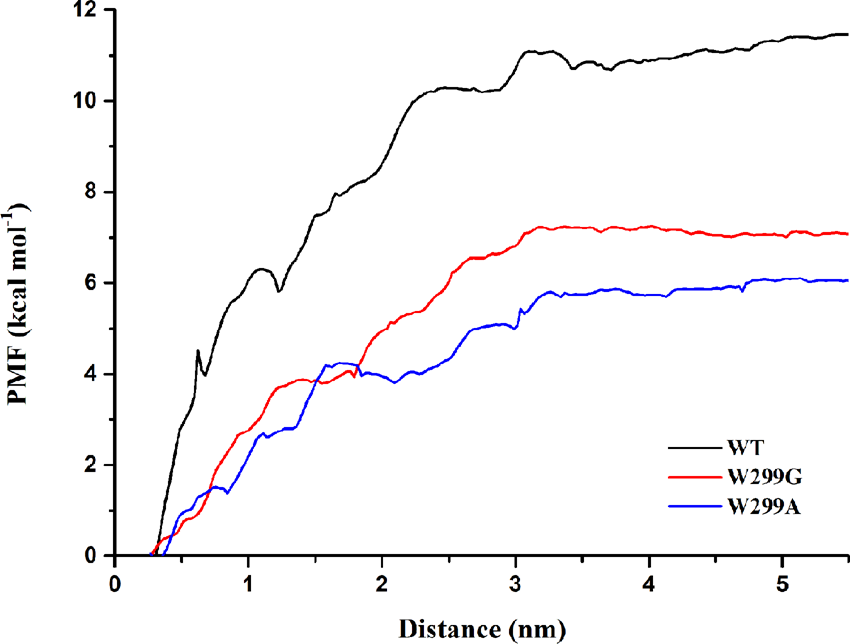


**Fig. S10** Potential of mean force (PMF) profiles of the KsdD3 wild type and mutants over the distance along the substrate channel.
